# Association between the bed-to-nurse ratio and 30-day post-discharge mortality in patients undergoing surgery: a cross-sectional analysis using Korean administrative data

**DOI:** 10.1186/s12912-020-0410-7

**Published:** 2020-03-17

**Authors:** Yunmi Kim, Hyun-Young Kim, Eunyoung Cho

**Affiliations:** 1 College of Nursing, Eulji University, 553 Sanseong-daero, Sujeong-gu, Seongnam-si, 13135 Republic of Korea; 2 Department of Nursing, Jeonju University, 303 Cheonjam-ro, Wansan-gu, Jeonju-si, 55069 Republic of Korea; 3 Department of Nursing, Dongseo University, 47 Jurye-ro, Saha-gu, Busan, 47011 Republic of Korea

**Keywords:** Bed-to-nurse ratio, Mortality, Post-discharge, Surgery

## Abstract

**Background:**

The likelihood of inpatient mortality has been found to be reduced by increased nurse staffing in several settings, including general wards, emergency departments, and intensive care units. However, less research has investigated cases where patients die in the community setting due to a health problem that occurred after they were discharged post-surgery, because it is difficult to integrate hospital data and local community data. Therefore, this study investigated the association between the bed-to-nurse ratio and 30-day post-discharge mortality in patients undergoing surgery using national administrative data.

**Methods:**

The study analyzed data from 129,923 patients who underwent surgery between January 2014 and December 2015. The bed-to-nurse ratio was categorized as level 1 (less than 2.5), level 2 (2.5–3.4), level 3 (3.5–4.4), and level 4 (4.5 or greater). The chi-square test and GEE logistic regression analyses were used to explore the association between the bed-to-nurse ratio and 30-day post-discharge mortality.

**Results:**

1355 (0.01%) patients died within 30 days post-discharge. The 30-day post-discharge mortality rate in hospitals with a level 4 was 2.5%, representing a statistically significant difference from the rates of 0.8, 2 and 1.8% in hospitals with level 1, level 2, and level 3 staffing, respectively. In addition, the death rate was significantly lower at hospitals with a level 1 (OR = 0.62) or level 2 (OR = 0.63) bed-to-nurse ratio, using level 4 as reference.

**Conclusion:**

The results of this study are highly meaningful in that they underscore the necessity of in-hospital discharge nursing and continued post-discharge nursing care as a way to reduce post-discharge mortality risk. Furthermore, the relationship between nurse staffing levels and 30-day post-discharge mortality implies the need for a greater focus on discharge education. Policies are required to achieve proper nurse staffing levels in Korea, and thereby to enhance patient outcomes.

## Background

Surgery is an invasive process in which various human body parts undergo incision, removal, prosthesis application, and/or suturing, depending on the procedure. Therefore, surgery is accompanied by a high risk of complications such as gastrointestinal problems and complications, wound disclosure and surgical infection, venous thromboembolism, and failure to thrive or malnutrition. Due to the high rate of postoperative complications, 11.3–11.9% of patients who have undergone general surgery, craniotomy, or vascular surgery are readmitted within 30 days after discharge [1–3]. Several studies have investigated the 30-day outcomes of patients who have been discharged after surgery and found factors that could be targeted to improve those outcomes; in particular, nurse staffing is receiving attention as an important related variable [4].

Studies of the associations between inpatient or post-discharge patient mortality and nurse staffing for patients with various conditions—treated medically or surgically—have been conducted in many countries, and the likelihood of patient mortality has been found to be reduced by increased nurse staffing in several settings, including general wards, emergency departments, and intensive care units (ICU) [5–8]. Furthermore, in a Korean study of patients with diseases such as stroke and cardiovascular disease, it was found that in-hospital or 30-day mortality had statistically significant associations with the nurse staffing level [9–11]. Although many studies have investigated these patient outcomes, less research has investigated cases where patients die in the community setting, such as at home or a nursing home without appropriate safeguards, due to a health problem that occurred after they were discharged post-surgery. This gap in the literature exists because it is difficult to integrate hospital data and local community data. For this reason, based on previous studies showing associations between patient mortality and nurse staffing, this study expands the existing evidence by linking nurse staffing to post-discharge death in patients who underwent surgery.

The goal of nursing is to conduct safe and high-quality care for patients, and discharge education for self-care of patients at home after discharge is a core nursing process. That is why insufficient discharge preparation by nurses during admission makes it difficult for patients and their families to carry out treatment instructions and self-care for surgical wounds, and to find and link appropriate supporting resources [12], and can even cause readmission [13, 14]. To improve post-discharge patient outcomes, studies have been conducted to develop nurse-led case management or nurse-led transitional care programs and to measure the effects of enhancing discharge education while patients are hospitalized or of improving linkage with post-discharge home care; these programs have shown positive outcomes, such as reduction of the readmission rate, improvements in self-efficacy and quality of life, and cost-saving [15, 16]. Previous studies have empirically shown that discharge nursing should be done while surgical patients are admitted as inpatients and that nursing should be linked to home nursing after discharge to reduce post-discharge readmission and mortality of surgical patients. The purpose of the present study was to evaluate the impact of increasing nurse staffing on improving patient outcomes, with potential policy implications.

## Methods

This was a cross-sectional study using a secondary analysis of National Health Insurance Service (NHIS) data from January 2014 to December 2015 to examine the association between the bed-to-nurse ratio and death within 30 days after discharge among patients who underwent surgery.

### Setting and participants

This cross-sectional, retrospective study was performed using NHIS data on 129,923 patients. The NHIS is a nationwide, comprehensive health insurance system that is administered by the government of Korea. The NHIS manages all fee-for-service claim data, which encompass patients’ medical history from all medical institutions. We extracted patient-level and hospital-level data for this study. The inclusion criteria for patients in this study were patients aged between 20 and 85 who underwent 12 types of surgery between January 2014 and December 2015. The 12 types of surgery analyzed in this study were determined using Korean Diagnosis-Related Group (KDRG) categories with relatively high death rates according to previous studies (craniotomy, cardiovascular surgery, digestive system surgery, and hepatobiliary system surgery). Their KDRG categories were as follows: major craniotomy except for trauma (B011-B019), other craniotomy except for trauma (B021-B022), craniotomy for trauma (B030), cardiac valve procedure with cardiac catheter (F021-F023), cardiac valve procedure without cardiac catheter (F031-F033), coronary bypass with/without cardiac catheter (F041-F044), major reconstructive vascular procedure (F061-F064), amputation for circulatory system disorders (F120), esophageal procedure (G011-G013), major small and large bowel procedure (G031-G033), stomach and duodenal procedure (G041-G046), and pancreas, liver and shunt procedure (H011-H016) [1–3, 17]. The exclusion criteria encompassed patients who had been transferred to other hospitals during their hospital stay after surgery, inpatients in special treatment rooms (aseptic rooms, isolated rooms, rooms with lead shielding, etc.), hospice patients, inpatients for less than 1 day, or inpatients who were admitted for medical treatment/care due to official duties or injuries inflicted by a third party. Initially, according to these criteria, 138,318 patients without missing data were extracted and the dataset was constructed by adding personal information, as well as general information. In addition, data from 578 hospitals that performed these patients’ surgery were merged.

Overall, 129,923 surgical patients from 214 hospitals were included in the analysis, after the further exclusion of 4822 patients who died in-hospital, 1663 patients who were readmitted within 30 days, and 1972 patients who underwent surgery at small hospitals with less than the minimum patient volume (fewer than 24 discharged patients during the study period) were then excluded to obtain reliable estimates from the statistical analysis [11, 18]. The study subjects were selected according to the process presented in Supplement 1.

### Variables

The outcome variable was death within 30 days after discharge to home from a hospital. The death was confirmed by integrating the data of 129,923 patients extracted from the NHIS data and death registry data of Statistics Korea using temporary IDs.

The bed-to-nurse ratio of the general wards, the primary independent variable of interest, calculated by the number of total general ward beds divided by the number of nurses participating in the nursing care of the patients using those beds [5]. In this study, the bed-to-nurse ratios were extracted from the Adjusted Differentiated Inpatient Nursing Fees by staffing grades of the NHIS, which were implemented in November 1999 for the purpose of improving nurse staffing levels through financial incentives for institutions [11, 19]. Each hospital should notify the Health Insurance and Review Assessment Service of the bed-to-nurse ratio for general wards, adult/pediatric intensive care units, and neonatal intensive care units every 3 months. The bed-to-nurse ratio of general wards is calculated as the mean number of beds over 3 months divided by the mean number of full-time-equivalent nurses in the ward over the same period [20]. We categorized the bed-to-nurse ratio of general wards as level 1 (less than 2.5), level 2 (2.5–3.4), level 3 (3.5–4.4), and level 4 (4.5 or greater), applying the legal standards for nurse staffing levels in accordance with a previous study. The level 1 (< 2.5) hospitals adhered to the legally required nurse staffing level [19].

The following variables related to characteristics of hospitals were controlled for: hospital type (tertiary hospital or secondary hospital) based on medical laws, hospital ownership (public sector, educational foundation, medical or other corporation, or private corporation), hospital location (Seoul, metropolitan cities, or small and medium-sized cities), and bed-to-physician ratio.

The following additional patient-related variables were controlled for: age, gender, complications or comorbidities (CCs), type of health insurance (self-employed beneficiaries, self-employed dependents, employed beneficiaries, employed dependents, Medical Aid beneficiaries, and Medical Aid dependents), and admission route (emergency department, outpatient department). CCs were classified as 0 (no CCs), 1 (minor CCs), 2 (moderate CCs), and 3 (severe CCs) based on the KDRG Grouper, applying the complication and comorbidity levels (CCLs) of the Australian Refined Diagnosis Related Groups (ARDRGs). The CCLs provide a weighting of case complexity using integer values between 0 and 4 for all diagnoses; they make it possible to consider both already-present diagnoses at the time of admission (comorbidities) and acquired diagnoses after the patient’s admission (complications) when evaluating post-discharge mortality [21].

### Statistical analysis

In the statistical analyses, the *χ*^*2*^ test was used to assess differences in categorical variables (hospital characteristics and death or survival of patients). Generalized estimating equation (GEE) logistic regression was used to calculate the adjusted odds ratios (ORs) for 30-day post-discharge mortality, controlling for confounding variables (hospital type, hospital ownership, hospital location, bed-to-physician ratios, age, gender, type of surgery, CCs, type of health insurance, and admission route). As there was a correlation between subjects who treated in the same hospital due to the information about the hospital being the same, GEE logistic regression was used, because it has the advantage of correcting for the standard error when estimating parameters. Data were analyzed using SAS 9.4 (SAS Institute, Cary, NC, USA). Significance was set at *P* < .05.

## Results

### Hospital characteristics

The characteristics of the hospitals included in this study are presented in Table 1. The 214 hospitals comprised 20.1% tertiary hospitals and 79.9% general hospitals. Furthermore, 10.3, 27.1, 42.5, and 20.1% of the hospitals were owned by the public sector, educational foundations, medical or other corporations, and private corporations, respectively. The nurse staffing, defined based on the bed-to-nurse ratio, was level 1 (less than 2.5) in 29 (13.6%) hospitals, level 2 (2.5–3.4) in 115 (53.7%) hospitals, level 3 (3.5–4.4) in 30 (14.0%) hospitals, and level 4 (4.5 or greater) in 40 (18.7%) hospitals.

**Table 1:** Hospital characteristics (*n* = 214)

Variable	Categories	Total n (%)
Hospital type	Tertiary hospital	43 (20.1)
	Secondary hospital	171 (79.9)
Hospital ownership	Public sector	22 (10.3)
	Educational foundation	58 (27.1)
	Medical or other corporation	91 (42.5)
	Private corporation	43 (20.1)
Hospital location	Seoul	48 (22.4)
	Metropolitan cities	62 (29.0)
	Small and medium-sized cities	104 (48.6)
Bed-to- physician ratio	Less than 2.0	14 (6.5)
	2.0–3.9	78 (36.5)
	4.0–5.9	32 (15.0)
	Over 6.0	90 (42.1)
	Mean ± SD^a^	6.3 ± 5.0
Bed-to-nurse ratio	Level 1 (less than 2.5)	29 (13.6)
	Level 2 (2.5–3.4)	115 (53.7)
	Level 3 (3.5–4.4)	30 (14.0)
	Level 4 (4.5 or greater)	40 (18.7)

^a^Mean ± SD: mean ± standard deviation, used in GEE logistic regression for confounding variables

### Patients’ characteristics and differences between the patients who died or survived within 30 days after discharge

Patients’ overall characteristics and differences between the patients who died and survived are presented in Table 2 and Table 3. Among the hospital characteristics, significant differences were found for hospital type (*χ*^*2*^ = 43.33, *P <* .001), hospital ownership (*χ*^*2*^ = 19.62, *P <* .001), hospital location (*χ*^*2*^ = 65.80, *P <* .001), bed-to-physician ratio (*χ*^*2*^ = 64.74, *P <* .001), and the bed-to-nurse ratio of general wards (*χ*^*2*^ = 1 13.91, *P <* .001) (Table 1). The 30-day post-discharge mortality rate in hospitals with a level 4 bed-to-nurse ratio (4.5 or greater) was 2.5%, representing a statistically significant difference from the rates of 0.8, 2 and 1.8% in hospitals with level 1 (less than 2.5), level 2 (2.5–3.5), and level 3 (3.5–4.5) staffing, respectively (Table 2).

**Table 2:** Differences between patients who died or survived within 30 days after discharge by hospital characteristics (*n* = 129,923)

Variable	Categories	Total n (%)	Died (*n* = 1355, 0.01%) n (%)	Survived (*n* = 128,568, 99.9%) n (%)	*χ*^*2*^	*p*
Hospital type	Tertiary hospital	89,783 (69.1)	825 (0.9)	88,958 (99.1)	43.33	< .001
	Secondary hospital	40,140 (30.9)	530 (1.3)	39,610 (98.7)		
Hospital ownership	Public sector	24,717 (19.0)	218 (0.9)	24,499 (99.1)	19.62	< .001
	Educational foundation	54,464 (41.9)	636 (1.2)	53,828 (98.8)		
	Medical or other corporation	47,036 (36.2)	453 (1.0)	46,583 (99.0)		
	Private corporation	3706 (2.9)	48 (1.3)	3658 (98.7)		
Hospital location	Seoul	58,146 (44.8)	460 (0.8)	57,686 (99.2)	65.80	< .001
	Metropolitan city	31,712 (24.4)	381 (1.2)	31,331 (98.8)		
	Small and medium sized city	40,065 (30.8)	514 (1.3)	39,551 (98.7)		
Bed-to-physician ratios	Less than 2.0	50,498 (38.9)	358 (0.7)	50,140 (99.3)	150.43	< .001
	2.0–3.9	65,116 (50.1)	761 (1.2)	64,355 (98.8)		
	4.0–5.9	7087 (5.5)	81 (1.1)	7006 (98.9)		
	6.0 or greater	7222 (5.6)	155 (2.2)	7067 (97.9)		
Bed-to-nurse ratios	Level 1 (Less than 2.5)	71,087 (54.7)	588 (0.8)	70,499 (99.2)	113.91	< .001
	Level 2 (2.5–3.4)	54,681 (42.1)	674 (1.2)	54,007 (98.8)		
	Level 3 (3.5–4.4)	1483 (1.1)	26 (1.8)	1457 (98.3)		
	Level 4 (4.5 or greater)	2672 (2.1)	67 (2.5)	2605 (97.5)

**Table 3:** Differences between patients who died or survived within 30 days after discharge by patient characteristics (*n* = 129,923)

Variable	Categories	Total n (%)	Died (*n* = 1355, 0.01%) n (%)	Survived (*n* = 128,568, 99.9%) n (%)	*χ*^*2*^	*p*
Age, years	Less than 30	2564 (2.0)	12 (0.5)	2552 (99.5)	411.29	< .001
	30–39	6707 (5.2)	35 (0.5)	6672 (99.5)		
	40–49	18,083 (13.9)	86 (0.5)	17,997 (99.5)		
	50–59	35,152 (27.1)	244 (0.7)	34,908 (99.3)		
	60–69	34,152 (26.3)	319 (0.9)	33,833 (99.1)		
	70 or greater	33,265 (25.6)	659 (2.0)	32,606 (98.0)		
	Mean ± SD^a^	59.6 ± 12.9				
Gender	Male	78,485 (60.4)	859 (1.1)	77,626 (98.9)	5.10	.024
	Female	51,438 (39.6)	496 (1.0)	50,942 (99.0)		
Type of surgery	Major craniotomy except for trauma	29,898 (23.0)	417 (1.4)	29,481 (98.6)	414.92	< .001
	Other craniotomy except for trauma	6586 (5.1)	176 (2.7)	6410 (97.3)		
	Craniotomy for trauma	8683 (6.7)	171 (2.0)	8512 (98.0)		
	Cardiac valve procedure with cardiac catheter	2110 (1.6)	19 (0.9)	2091 (99.1)		
	Cardiac valve procedure without cardiac catheter	3506 (2.7)	22 (0.6)	3484 (99.4)		
	Coronary bypass with/without cardiac catheter	4079 (3.1)	31 (0.8)	4048 (99.2)		
	Major reconstructive vascular procedure	2811 (2.2)	45 (1.6)	2766 (98.4)		
	Amputation for circulatory system disorders	1121 (0.9)	17 (1.5)	1104 (98.5)		
	Esophageal procedure	2086 (1.6)	19 (0.9)	2067 (99.1)		
	Major small and large bowel procedure	12,401 (9.5)	56 (0.5)	12,345 (99.6)		
	Stomach and duodenal procedure	38,014 (29.3)	261 (0.7)	37,753 (99.3)		
	Pancreas, liver and shunt procedure	18,628 (14.3)	121 (0.7)	18,507 (99.4)		
Comorbidities or complications	None	74,365 (57.2)	357 (0.5)	74,008 (99.5)	618.31	< .001
	Minor	33,988 (26.2)	555 (1.6)	33,433 (98.4)		
	Moderate	17,297 (13.3)	402 (2.3)	16,895 (97.7)		
	Severe	4273 (3.3)	41 (1.0)	4232 (99.0)		
Type of health insurance	Self-employed beneficiary	27,507 (21.2)	256 (0.9)	27,251 (99.1)	174.70	< .001
	Self-employed dependent	12,803 (9.9)	148 (1.2)	12,655 (98.8)		
	Employed beneficiary	27,457 (21.1)	131 (0.5)	27,326 (99.5)		
	Employed dependent	54,718 (42.1)	671 (1.2)	54,047 (98.8)		
	Medical Aid beneficiary	6563 (5.1)	133 (2.0)	6430 (98.0)		
	Medical Aid dependent	875 (0.7)	16 (1.8)	859 (98.2)		
Admission route	Emergency department	101,516 (78.1)	730 (2.6)	27,677 (97.4)	821.25	< .001
	Outpatient department	28,407 (21.9)	625 (0.6)	100,891 (99.4)		

^a^Mean ± SD: mean ± standard deviation, used in GEE logistic regression for confounding variables

In addition, among the patient characteristics, significant differences were found for age (*χ*^*2*^ = 411.29, *P* ≤ .001), gender (*χ*^*2*^ = 5.10, *P* = .024), type of surgery (*χ*^*2*^ = 414.92, *P* ≤ .001), CCs (*χ*^*2*^ = 618.31, *P* ≤ .001), type of health insurance (*χ*^*2*^ = 174.70, *P* ≤ .001), and admission route (*χ*^*2*^ = 821.25, *P* ≤ .001) (Table 3).

### Associations between nurse staffing level and post-discharge mortality

We next calculated the odds ratios (ORs) for post-discharge mortality, as shown in Table 4. Compared to a level 4 bed-to-nurse ratio (4.5 or greater), level 1 (less than 2.5) was associated with a significantly lower risk of death (OR = 0.62; 95% CI = 0.39–0.99), as was level 2 (2.5–3.4), with an OR of 0.63 (95% CI = 0.42–0.94).

**Table 4:** Hospital and patient variables as predictors of 30-day mortality after discharge across all patients (*n* = 129,923)

Variable	Categories	Odds Ratio	95% Confidence Interval	*p*
Hospital variables				
Hospital type	Tertiary hospital	1.25	0.98–1.58	.067
	Secondary hospital	1.00		
Hospital ownership	Public sector	1.06	0.72–1.58	.764
	Educational foundation	1.34	0.92–1.96	.124
	Medical or other corporation	1.27	0.88–1.82	.197
	Private corporation	1.00		
Hospital location	Seoul	0.98	0.78–1.23	.851
	Metropolitan cities	0.95	0.79–1.14	.600
	Small and medium-sized cities	1.00		
Bed-to-physician ratio		1.02	0.99–1.05	.189
Bed-to-nurse ratios	Level 1 (Less than 2.5)	0.62	0.39–0.99	.044
	Level 2 (2.5–3.4)	0.63	0.42–0.94	.023
	Level 3 (3.5–4.4)	0.73	0.44–1.21	.219
	Level 4 (4.5 or greater)	1.00		
Patient variables				
Age, years		1.04	1.04–1.05	< .001
Sex	Male	1.40	1.22–1.60	< .001
	Female	1.00		
Type of surgery	Major craniotomy except for trauma	1.66	1.21–2.28	.002
	Other craniotomy except for trauma	1.62	1.15–2.27	.005
	Craniotomy for trauma	0.73	0.53–1.03	.072
	Cardiac valve procedure with cardiac catheter	0.76	0.43–1.33	.334
	Cardiac valve procedure without cardiac catheter	0.64	0.36–1.13	.123
	Coronary bypass with/without cardiac catheter	0.66	0.41–1.04	.076
	Major reconstructive vascular procedure	2.40	1.46–3.93	.001
	Amputation for circulatory system disorders	1.30	0.76–2.24	.342
	Esophageal procedure	1.24	0.74–2.08	.410
	Major small and large bowel procedure	0.42	0.29–0.62	< .001
	Stomach and duodenal procedure	0.70	0.53–0.93	.015
	Pancreas, liver and shunt procedure	1.00		
Comorbidities or complications	None	0.38	0.26–0.55	< .001
	Minor	1.09	0.77–1.53	.637
	Moderate	1.57	1.12–2.19	.009
	Severe	1.00		
Type of health insurance	Self-employed beneficiary	0.53	0.32–0.89	.017
	Self-employed dependent	0.84	0.48–1.48	.554
	Employed beneficiary	0.43	0.25–0.72	.002
	Employed dependent	0.62	0.37–1.04	.069
	Medical Aid beneficiary	0.97	0.54–1.77	.929
	Medical Aid dependent	1.00		
Admission route	Emergency department	2.73	2.33–3.21	< .001
	Outpatient department	1.00		

For every 1-year increase in patients’ age, the mortality rate was found to increase by 4% (OR = 1.04; 95% CI = 1.04–1.05). Males had a significantly higher risk of mortality than females, with an OR of 1.40 (95% CI = 1.22–1.60). The ORs showed statistically significant differences depending on surgery type. In terms of type of surgery, patients who underwent major craniotomy except for trauma were found to have an OR for mortality of 1.66 (95% CI = 1.21–2.28). Other craniotomy except for trauma was associated with an OR of 1.62 (95% CI = 1.15–2.27), major reconstructive vascular procedure with OR of 2.40 (95% CI = 1.46–3.93), major small and large bowel procedure with an OR of 0.42 (95% CI = 0.29–0.62), and stomach and duodenal procedure with an OR of 0.70 (95% CI = 0.53–0.93), using pancreas, liver and shunt procedures as the reference category.

Patient with no CCs were found to have an OR for mortality of 0.38 (95% CI = 0.26–0.55) and those with moderate CCs had an OR of 1.57 (95% CI = 1.12–2.19) compared to patients with severe CC. In terms of type of health insurance, self-employed beneficiaries had an OR for mortality of 0.53 (95% CI = 0.32–0.89), and employed beneficiaries had an OR of 0.43 (95% CI = 0.25–0.72) compared to Medical Aid dependents. The OR for the emergency department admission route was 2.73 (95% CI = 2.33–3.21) compared to admission via an outpatient department.

## Discussion

### Bed-to-nurse ratio and 30-day post-discharge mortality

The purpose of the present study was to investigate the association between nurse staffing levels of hospitals and 30-day mortality post-discharge in Korea. The study showed that 1355 patients died within 30 days after discharge, and that higher nurse staffing levels were associated with significantly lower ORs for mortality. More specifically, bed-to-nurse ratios of less than 2.5 and 2.5–3.4 were associated with 30-day post-discharge mortality probabilities that were 38% (OR = 0.62, 95% CI = 0.39–0.99) and 37% (OR = 0.63, 95% CI = 0.42–0.94) lower, respectively, than that of a bed-to-nurse ratio of 4.5 or over. This result is similar to that of a previous study conducted in hospitals in Korea, which showed that when the nurse staffing standard of the Adjusted Differentiated Inpatient Nursing Fees by staffing grades of the NHIS (the same as used in this study) was applied, patients who underwent surgery in hospitals with a nurse staffing grade of 2–3 (bed-to-nurse ratio 2.5–3.4), 4–5 (bed-to-nurse ratio 3.5–4.4), and 6–7 (bed-to-nurse ratio 4.5 or greater) had 57, 78, and 199% higher likelihoods of in-hospital mortality, respectively, than patients who underwent surgery in hospitals with a nurse staffing grade of 0–1 (bed-to-nurse ratio less than 2.5) [17]. On this basis, it could be inferred that the influence of the nurse staffing level in hospitals on the health outcomes of surgical patients extends to the post-discharge period, as well as the in-hospital period.

In this study, nurse staffing was the only hospital-related variable that showed a significant association with post-discharge mortality of surgical patients. Although it is difficult to find studies that allow direct comparisons with the present study, as not many studies have been conducted on the association between nurse staffing and post-discharge mortality, the results of this study are similar to those of studies that analyzed in-hospital or 30-day mortality. In a study conducted among patients who received treatment in an ICU due to cardiovascular disease, when the ICU staffing level of general hospitals gradually increased from 0.5 or below to 2.0 or above, 30-day mortality after discharge increased significantly by 37–50% [9]. In a study of mortality in stroke patients with stroke, a similar association was found for nurse-to-patient ratios in general wards and the ICU. In addition, compared with in-hospital mortality, 30-day mortality had a more obvious increase as nurse staffing became worse [11]. As such, in studies conducted in Korea, nurse staffing has consistently been found to be associated with multiple measures of mortality. Studies on nurse staffing and in-hospital deaths in other countries revealed associations between higher staffing levels of registered nurses and lower patient mortality rates [22, 23]. Therefore, if an appropriate level of nurse staffing is secured, the mortality rate of patients could be expected to decrease.

In previous studies, the variable that mediated the relationship between nurse staffing and the patient outcome of death was inferred to be an excessive workload and missed nursing care [4, 11], in light of the finding that when the mean number of patients per nurse providing direct care was 8.3, missed nursing care (including preparing patients and families for discharge and educating patients and family) was 25.6%, and when missed care by nurses increased by 10%, the 30-day mortality increased by 16% [4]. In a Korean study, based on the Adjusted Differentiated Inpatient Nursing Fees by staffing grades of ICUs, basic nursing care was 5.27 times higher and 30-day mortality was 0.13 times lower for institutions with a grade of 1–3 (the best) compared to those with a grade of 8–9 (the worst) [11]. Based on that study, the need was identified to investigate the status of basic nursing care at the time of discharge, and the most unmet need for post-discharge nursing care was found to be counseling, followed by equipment operation and instructions [12]. A systematic review on associations between nurse staffing and omissions in nursing care found low nurse staffing to be associated with missed nursing care in hospitals, and ensuring patient discharge planning was one of the types of nursing care that was omitted [24]. Discharge education that includes such content is a key mechanism for delivering necessary knowledge through individualized instruction regarding post-discharge care [25, 26], and programs such as discharge-transfer interventions, nurse-led case management, and nurse-led home-based case management have been suggested in previous international studies. It is also important to investigate which discharge education program is delivered by whom, how, and when. Those programs should be regularly implemented many times during patients’ hospital stay from admission and up to 6 months after discharge [16, 27]. In international studies, discharge planning programs in hospitals have been implemented by nurses with the job titles of ‘discharge planning nurse,’ ‘nurse discharge advocate,’ or ‘nurse case manager’ [15, 16, 28]. In this context, it is noteworthy that a Korean study analyzing 30-day mortality after discharge showed that nurse staffing had a significant effect on 30-day mortality after discharge, while the number of doctors did not [9].

Although the nurse-to-patient ratio is an important variable affecting patient mortality, the requirement of two nurses per five patients specified in the Medical Service Act of Korea is a relatively low nurse staffing standard compared to other countries [7]. Moreover, despite such a low standard, the percentage of secondary and tertiary hospitals complying with the Medical Service Act was 63% as of 2013 [29] and 32.3% of the hospitals (those with level 3 and 4 staffing in this study) did not comply with the law. There is a need for improvement in this regard.

This study classified the bed-to-nurse ratio by applying the Adjusted Differentiated Inpatient Nursing Fees by staffing grades, which sets the rate of operated sickbeds and nurses using an arithmetical standard. However, in other countries, research has been conducted based on the nursing skill mix. In a study relating to 30-day mortality after heart surgery, when the proportion of nurses with less than 2 years of ICU experience among nurses who worked in the ICU was over 1/3, mortality increased by 18% [6]. Additionally, the patient discharge readiness score of each nurse made a difference of 9.07% in patient discharge readiness scores and nurse productivity was found to be significantly associated with readmission probability [30].

Based on the issues discussed above, this study has the following implications. There is a need for research relating to economic analyses, such as ensuring appropriate nurse staffing levels and cost-benefit analyses of nursing-led discharge management programs, in order to reduce in-hospital and post-discharge mortality. Additionally, when making policy decisions, the period in which benefits occur should be extended after discharge instead of being limited to the hospital stay to obtain accurate and valid findings.

### Hospital and patient characteristics and 30-day post-discharge mortality

The major results related to the independent variables analyzed in this study are as follows. The variables that were significantly associated with 30-day post-discharge mortality were primarily general characteristics of the patients, and hospital characteristics—except for nurse staffing—did not show significant associations. These results are similar to those of a study that analyzed inpatient mortality [17]. The results are also in accordance with a study showing that the medical staff who had the greatest impact on patient death in the ICU were nurses; although the likelihood of mortality was decreased by 10% with higher numbers of nurses per bed, the number of support staff (administrative, clerical, technical, and scientific staff) had no significant association [31].

In a previous study, age was found to be associated with post-surgical mortality [6, 17], and the results of this study that the risk of mortality increased by 4% with every 1-year increment in age support those of previous research. In this study, males had a higher risk of mortality than females. This is in line with previous studies consistently showing that the mortality risk of males was higher than that of females, although some studies found this trend to be statistically significant, while others did not [9, 17].

In this study, moderate complications or comorbidities (CCs) were associated with a 57% higher risk of mortality than severe CCs. This is similar to the result of a previous Korean study on post-surgical inpatient mortality and complications, which found that moderate CCs were associated with an elevated risk [17]. In terms of type of insurance, the 30-day post-discharge mortality risk was highest in Medical Aid dependents and was lowest in workers who paid for National Health Insurance themselves. This is in accordance with previous findings that the ORs for unplanned readmission under Medicaid for some people with limited income and resources were higher than those of patients who received health coverage from health maintenance organizations in the USA [2] and that the ORs for in-hospital mortality among stroke patients with Medical Aid were higher for some people with limited income and resources in Korea [10]. Based on these findings, participants’ engagement in economic activities and social characteristics can be inferred to have a significant influence on post-discharge mortality and in-hospital mortality.

When the admission route was the emergency department, both post-discharge mortality and in-hospital mortality have been found to be higher [9, 10, 17], likely due to the increased acuity of patients admitted through this route. A previous study found that when patients had heart surgery after admission to the emergency department, being cared for by nurses with little experience was associated with a higher 30-day mortality rate, implying that the need for experts in the emergency department is high [6] and that unplanned admissions through the emergency department must be reduced by enhancing discharge education.

The major limitation of this study is related to the measurement of nurse staffing levels. International studies have measured nurse staffing levels in various ways, such nursing hours per patient day, nurse staffing by shift or the occupancy rate, and the education level of nurses [18, 23, 32]. However, this study only used the bed-to-nurse ratio because information on other indices was unavailable in the data used in this study; furthermore, this study could not control for the individual characteristics of nursing organizations and nurses due to limitations in the data. Another limitation is that differences in discharge nursing care according to the nurse staffing level were not directly measured; instead, we referenced the results of previous studies regarding this point. Further research should examine missed nursing discharge care according to the nurse staffing level. A final limitation of this study is that definitive conclusions regarding causal relationships cannot be drawn due to the cross-sectional nature of the study design. Despite these limitations, the results of this study are highly meaningful in that they underscore the necessity of in-hospital discharge nursing and continued post-discharge nursing care as a way to reduce post-discharge mortality risk. In addition, we have improved the generalizability of the findings by using national administrative data, unlike most previous studies, which were conducted using data from only certain regions or hospitals.

## Conclusions

This study investigated the association between nurse staffing in Korean hospitals and the mortality risk of patients who had been discharged after receiving surgery and were not readmitted. Patients were analyzed by linking National Health Insurance data with the annual death statistics from Statistics Korea. This study has significant meaning in that it confirmed that an adequate nurse staffing level was significantly associated with 30-day post-discharge mortality among surgical patients after controlling for hospital characteristics.

## Supplementary information

**Additional file 1:** Supplement 1. Flow chart of patient selection based on the National Health Insurance Service data.

## Data Availability

The datasets generated from the National Health Insurance Service (NHIS) data. However, the dataset are not public available due the data is only available to persons authorized by NHIS.

## Abbreviations

CCs: Complications and comorbidities
GEE: Generalized estimating equation
KDRG: Korean Diagnosis-Related Group
NHIS: National Health Insurance Service
OR: Odds ratio
SD: Standard deviation
