# Supplementary material for: Association between the bed-to-nurse ratio and 30-day post-discharge mortality in patients undergoing surgery: a cross-sectional analysis using Korean administrative data
Source: BMC Nurs. 2020 Mar 17;19:17. doi: 10.1186/s12912-020-0410-7 (PMC7076936; doi:10.1186/s12912-020-0410-7)
Supplement: Supplementary file 1 — Additional file 1: Supplement 1. Flow chart of patient selection based on the National Health Insurance Service data. [file 12912_2020_410_MOESM1_ESM.pdf]

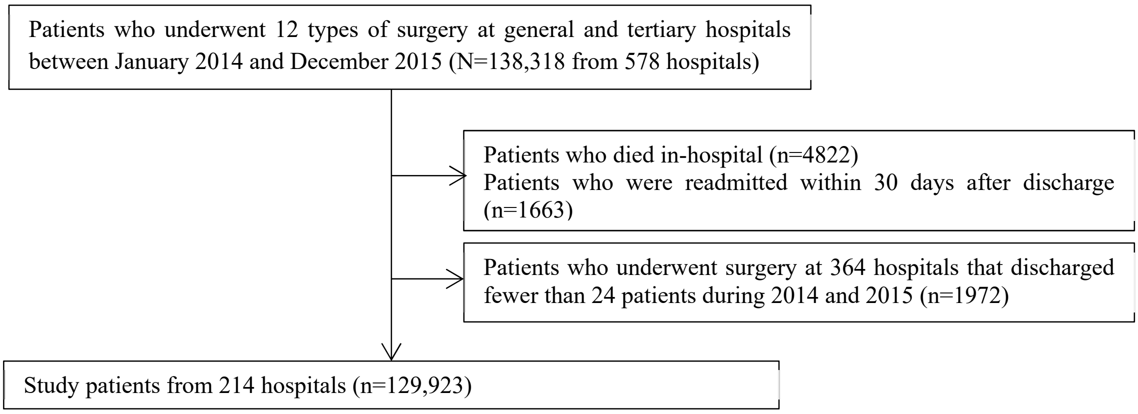

Supplement 1 Flow chart of patient selection based on the National Health Insurance Service data
